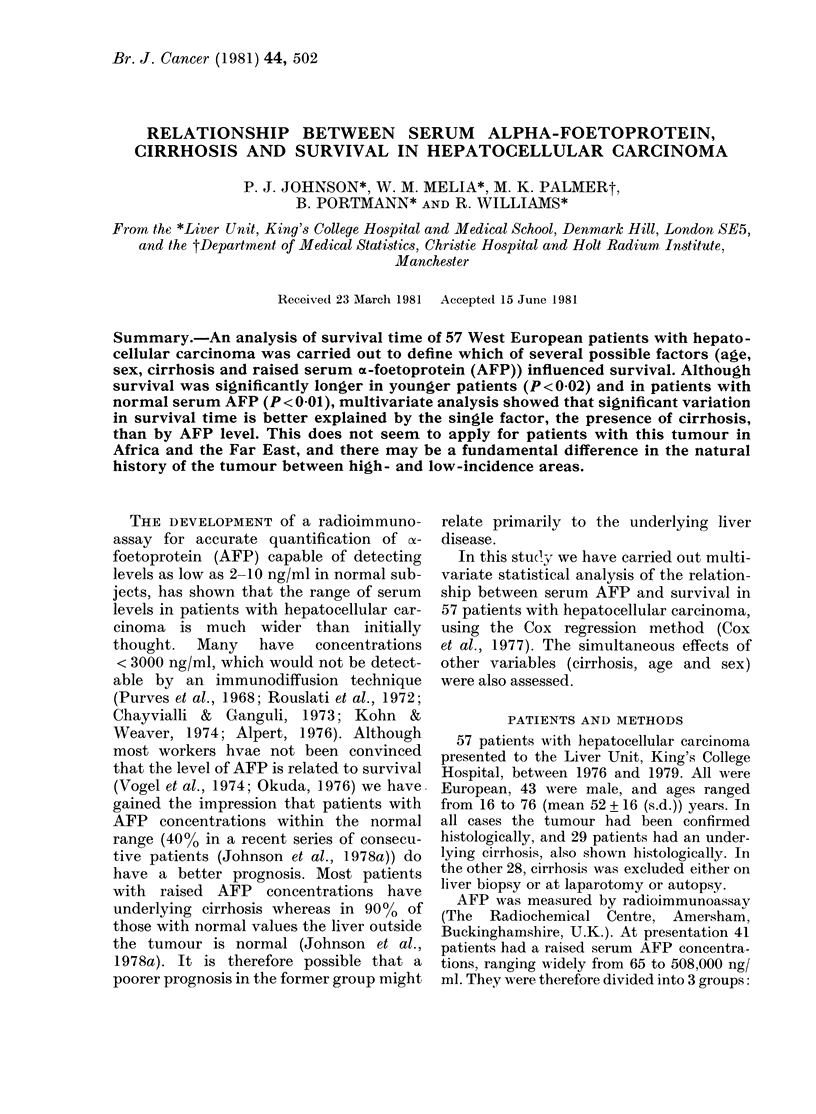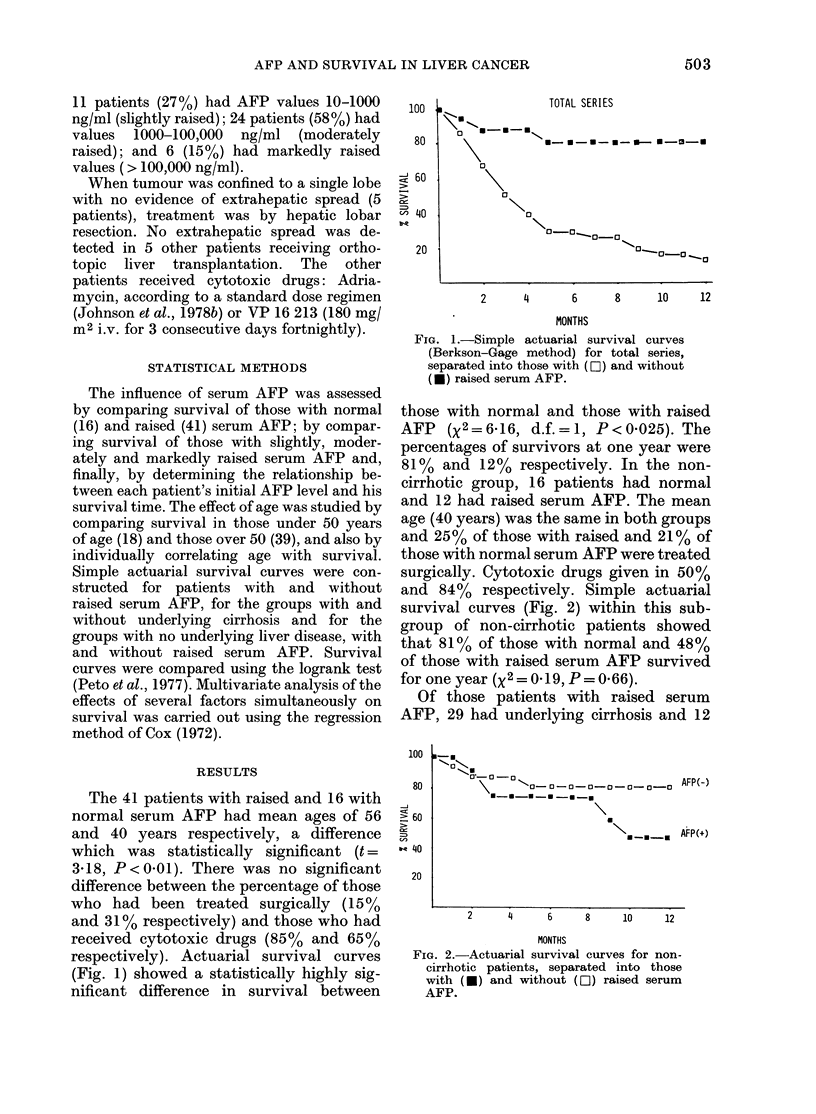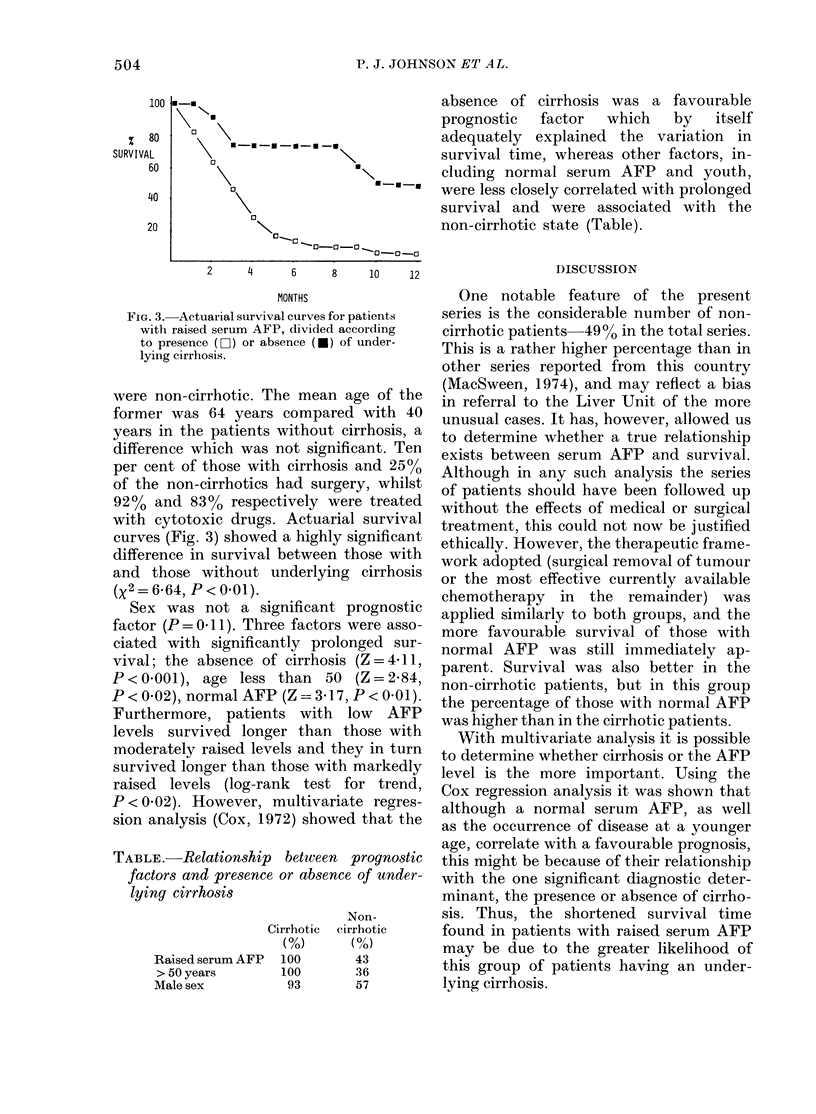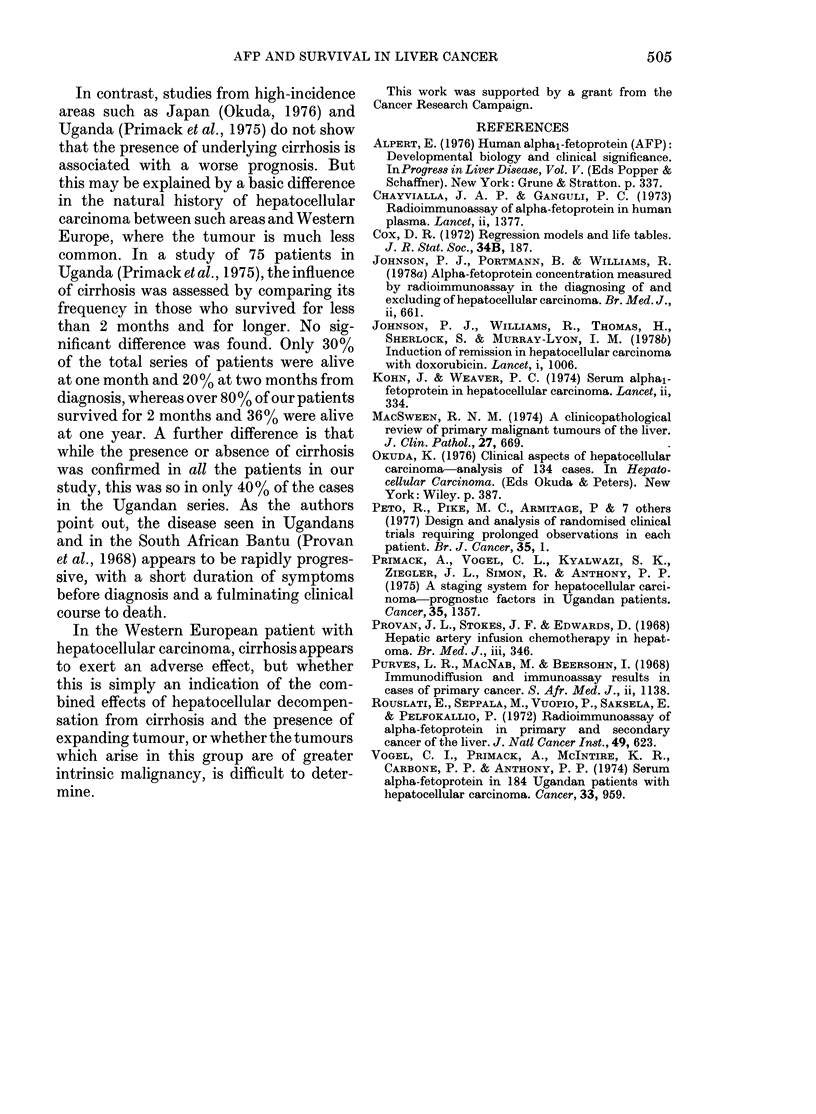# Relationship between serum alpha-foetoprotein, cirrhosis and survival in hepatocellular carcinoma.

**DOI:** 10.1038/bjc.1981.218

**Published:** 1981-10

**Authors:** P. J. Johnson, W. M. Melia, M. K. Palmer, B. Portmann, R. Williams

## Abstract

An analysis of survival time of 57 West European patients with hepatocellular carcinoma was carried out to define which of several possible factors (age, sex, cirrhosis and raised serum alpha-foetoprotein (AFP)) influenced survival. Although survival was significantly longer in younger patients (P less than 0.02) and in patients with normal serum AFP (P less than 0.01), multivariate analysis showed that significant variation in survival time is better explained by the single factor, the presence of cirrhosis, than by AFP level. This does not seem to apply for patients with this tumour in Africa and the Far East, and there may be a fundamental difference in the natural history of the tumour between high- and low-incidence areas.


*Br. J. Cancer* (1981) **44**, 502

### RELATIONSHIP BETWEEN SERUM ALPHA-FOETOPROTEIN, CIRRHOSIS AND SURVIVAL IN HEPATOCELLULAR CARCINOMA

P. J. JOHNSON*, W. M. MELIA*, M. K. PALMER†, B. PORTMANN* AND R. WILLIAMS*

*From the *Liver Unit, King's College Hospital and Medical School, Denmark Hill, London SE5, and the †Department of Medical Statistics, Christie Hospital and Holt Radium Institute, Manchester*

Received 23 March 1981 Accepted 15 June 1981

**Summary.—An analysis of survival time of 57 West European patients with hepatocellular carcinoma was carried out to define which of several possible factors (age, sex, cirrhosis and raised serum α-foetoprotein (AFP)) influenced survival. Although survival was significantly longer in younger patients ($P<0\cdot02$) and in patients with normal serum AFP ($P<0\cdot01$), multivariate analysis showed that significant variation in survival time is better explained by the single factor, the presence of cirrhosis, than by AFP level. This does not seem to apply for patients with this tumour in Africa and the Far East, and there may be a fundamental difference in the natural history of the tumour between high- and low-incidence areas.**

THE DEVELOPMENT of a radioimmunoassay for accurate quantification of α-foetoprotein (AFP) capable of detecting levels as low as 2–10 ng/ml in normal subjects, has shown that the range of serum levels in patients with hepatocellular carcinoma is much wider than initially thought. Many have concentrations < 3000 ng/ml, which would not be detectable by an immunodiffusion technique (Purves *et al.*, 1968; Rouslati *et al.*, 1972; Chayvialli & Ganguli, 1973; Kohn & Weaver, 1974; Alpert, 1976). Although most workers hvae not been convinced that the level of AFP is related to survival (Vogel *et al.*, 1974; Okuda, 1976) we have gained the impression that patients with AFP concentrations within the normal range (40% in a recent series of consecutive patients (Johnson *et al.*, 1978*a*)) do have a better prognosis. Most patients with raised AFP concentrations have underlying cirrhosis whereas in 90% of those with normal values the liver outside the tumour is normal (Johnson *et al.*, 1978*a*). It is therefore possible that a poorer prognosis in the former group might relate primarily to the underlying liver disease.

In this study we have carried out multivariate statistical analysis of the relationship between serum AFP and survival in 57 patients with hepatocellular carcinoma, using the Cox regression method (Cox *et al.*, 1977). The simultaneous effects of other variables (cirrhosis, age and sex) were also assessed.

### PATIENTS AND METHODS

57 patients with hepatocellular carcinoma presented to the Liver Unit, King's College Hospital, between 1976 and 1979. All were European, 43 were male, and ages ranged from 16 to 76 (mean $52 \pm 16$ (s.d.)) years. In all cases the tumour had been confirmed histologically, and 29 patients had an underlying cirrhosis, also shown histologically. In the other 28, cirrhosis was excluded either on liver biopsy or at laparotomy or autopsy.

AFP was measured by radioimmunoassay (The Radiochemical Centre, Amersham, Buckinghamshire, U.K.). At presentation 41 patients had a raised serum AFP concentrations, ranging widely from 65 to 508,000 ng/ml. They were therefore divided into 3 groups:

11 patients (27%) had AFP values 10–1000 ng/ml (slightly raised); 24 patients (58%) had values 1000–100,000 ng/ml (moderately raised); and 6 (15%) had markedly raised values (>100,000 ng/ml).

When tumour was confined to a single lobe with no evidence of extrahepatic spread (5 patients), treatment was by hepatic lobar resection. No extrahepatic spread was detected in 5 other patients receiving orthotopic liver transplantation. The other patients received cytotoxic drugs: Adriamycin, according to a standard dose regimen (Johnson *et al.*, 1978*b*) or VP 16 213 (180 mg/$m^2$ i.v. for 3 consecutive days fortnightly).

### STATISTICAL METHODS

The influence of serum AFP was assessed by comparing survival of those with normal (16) and raised (41) serum AFP; by comparing survival of those with slightly, moderately and markedly raised serum AFP and, finally, by determining the relationship between each patient's initial AFP level and his survival time. The effect of age was studied by comparing survival in those under 50 years of age (18) and those over 50 (39), and also by individually correlating age with survival. Simple actuarial survival curves were constructed for patients with and without raised serum AFP, for the groups with and without underlying cirrhosis and for the groups with no underlying liver disease, with and without raised serum AFP. Survival curves were compared using the logrank test (Peto *et al.*, 1977). Multivariate analysis of the effects of several factors simultaneously on survival was carried out using the regression method of Cox (1972).

### RESULTS

The 41 patients with raised and 16 with normal serum AFP had mean ages of 56 and 40 years respectively, a difference which was statistically significant ($t = 3{\cdot}18$, $P < 0{\cdot}01$). There was no significant difference between the percentage of those who had been treated surgically (15% and 31% respectively) and those who had received cytotoxic drugs (85% and 65% respectively). Actuarial survival curves (Fig. 1) showed a statistically highly significant difference in survival between

FIG. 1.—Simple actuarial survival curves (Berkson–Gage method) for total series, separated into those with (□) and without (■) raised serum AFP.

those with normal and those with raised AFP ($\chi^2 = 6{\cdot}16$, d.f. $= 1$, $P < 0{\cdot}025$). The percentages of survivors at one year were 81% and 12% respectively. In the non-cirrhotic group, 16 patients had normal and 12 had raised serum AFP. The mean age (40 years) was the same in both groups and 25% of those with raised and 21% of those with normal serum AFP were treated surgically. Cytotoxic drugs given in 50% and 84% respectively. Simple actuarial survival curves (Fig. 2) within this subgroup of non-cirrhotic patients showed that 81% of those with normal and 48% of those with raised serum AFP survived for one year ($\chi^2 = 0{\cdot}19$, $P = 0{\cdot}66$).

Of those patients with raised serum AFP, 29 had underlying cirrhosis and 12

FIG. 2.—Actuarial survival curves for non-cirrhotic patients, separated into those with (■) and without (□) raised serum AFP.

FIG. 3.—Actuarial survival curves for patients with raised serum AFP, divided according to presence (□) or absence (■) of underlying cirrhosis.

were non-cirrhotic. The mean age of the former was 64 years compared with 40 years in the patients without cirrhosis, a difference which was not significant. Ten per cent of those with cirrhosis and 25% of the non-cirrhotics had surgery, whilst 92% and 83% respectively were treated with cytotoxic drugs. Actuarial survival curves (Fig. 3) showed a highly significant difference in survival between those with and those without underlying cirrhosis ($\chi^2 = 6{\cdot}64$, $P < 0{\cdot}01$).

Sex was not a significant prognostic factor ($P = 0{\cdot}11$). Three factors were associated with significantly prolonged survival; the absence of cirrhosis ($Z = 4{\cdot}11$, $P < 0{\cdot}001$), age less than 50 ($Z = 2{\cdot}84$, $P < 0{\cdot}02$), normal AFP ($Z = 3{\cdot}17$, $P < 0{\cdot}01$). Furthermore, patients with low AFP levels survived longer than those with moderately raised levels and they in turn survived longer than those with markedly raised levels (log-rank test for trend, $P < 0{\cdot}02$). However, multivariate regression analysis (Cox, 1972) showed that the absence of cirrhosis was a favourable prognostic factor which by itself adequately explained the variation in survival time, whereas other factors, including normal serum AFP and youth, were less closely correlated with prolonged survival and were associated with the non-cirrhotic state (Table).

TABLE.—*Relationship between prognostic factors and presence or absence of underlying cirrhosis*

| | Cirrhotic (%) | Non-cirrhotic (%) |
|---|---|---|
| Raised serum AFP | 100 | 43 |
| > 50 years | 100 | 36 |
| Male sex | 93 | 57 |

### DISCUSSION

One notable feature of the present series is the considerable number of non-cirrhotic patients—49% in the total series. This is a rather higher percentage than in other series reported from this country (MacSween, 1974), and may reflect a bias in referral to the Liver Unit of the more unusual cases. It has, however, allowed us to determine whether a true relationship exists between serum AFP and survival. Although in any such analysis the series of patients should have been followed up without the effects of medical or surgical treatment, this could not now be justified ethically. However, the therapeutic framework adopted (surgical removal of tumour or the most effective currently available chemotherapy in the remainder) was applied similarly to both groups, and the more favourable survival of those with normal AFP was still immediately apparent. Survival was also better in the non-cirrhotic patients, but in this group the percentage of those with normal AFP was higher than in the cirrhotic patients.

With multivariate analysis it is possible to determine whether cirrhosis or the AFP level is the more important. Using the Cox regression analysis it was shown that although a normal serum AFP, as well as the occurrence of disease at a younger age, correlate with a favourable prognosis, this might be because of their relationship with the one significant diagnostic determinant, the presence or absence of cirrhosis. Thus, the shortened survival time found in patients with raised serum AFP may be due to the greater likelihood of this group of patients having an underlying cirrhosis.

In contrast, studies from high-incidence areas such as Japan (Okuda, 1976) and Uganda (Primack *et al.*, 1975) do not show that the presence of underlying cirrhosis is associated with a worse prognosis. But this may be explained by a basic difference in the natural history of hepatocellular carcinoma between such areas and Western Europe, where the tumour is much less common. In a study of 75 patients in Uganda (Primack *et al.*, 1975), the influence of cirrhosis was assessed by comparing its frequency in those who survived for less than 2 months and for longer. No significant difference was found. Only 30% of the total series of patients were alive at one month and 20% at two months from diagnosis, whereas over 80% of our patients survived for 2 months and 36% were alive at one year. A further difference is that while the presence or absence of cirrhosis was confirmed in *all* the patients in our study, this was so in only 40% of the cases in the Ugandan series. As the authors point out, the disease seen in Ugandans and in the South African Bantu (Provan *et al.*, 1968) appears to be rapidly progressive, with a short duration of symptoms before diagnosis and a fulminating clinical course to death.

In the Western European patient with hepatocellular carcinoma, cirrhosis appears to exert an adverse effect, but whether this is simply an indication of the combined effects of hepatocellular decompensation from cirrhosis and the presence of expanding tumour, or whether the tumours which arise in this group are of greater intrinsic malignancy, is difficult to determine.

This work was supported by a grant from the Cancer Research Campaign.

### REFERENCES

ALPERT, E. (1976) Human alpha$_1$-fetoprotein (AFP): Developmental biology and clinical significance. In *Progress in Liver Disease, Vol. V.* (Eds Popper & Schaffner). New York: Grune & Stratton. p. 337.

CHAYVIALLA, J. A. P. & GANGULI, P. C. (1973) Radioimmunoassay of alpha-fetoprotein in human plasma. *Lancet*, ii, 1377.

COX, D. R. (1972) Regression models and life tables. *J. R. Stat. Soc.*, **34B**, 187.

JOHNSON, P. J., PORTMANN, B. & WILLIAMS, R. (1978*a*) Alpha-fetoprotein concentration measured by radioimmunoassay in the diagnosing of and excluding of hepatocellular carcinoma. *Br. Med. J.*, ii, 661.

JOHNSON, P. J., WILLIAMS, R., THOMAS, H., SHERLOCK, S. & MURRAY-LYON, I. M. (1978*b*) Induction of remission in hepatocellular carcinoma with doxorubicin. *Lancet*, i, 1006.

KOHN, J. & WEAVER, P. C. (1974) Serum alpha$_1$-fetoprotein in hepatocellular carcinoma. *Lancet*, ii, 334.

MACSWEEN, R. N. M. (1974) A clinicopathological review of primary malignant tumours of the liver. *J. Clin. Pathol.*, **27**, 669.

OKUDA, K. (1976) Clinical aspects of hepatocellular carcinoma—analysis of 134 cases. In *Hepatocellular Carcinoma.* (Eds Okuda & Peters). New York: Wiley. p. 387.

PETO, R., PIKE, M. C., ARMITAGE, P & 7 others (1977) Design and analysis of randomised clinical trials requiring prolonged observations in each patient. *Br. J. Cancer*, **35**, 1.

PRIMACK, A., VOGEL, C. L., KYALWAZI, S. K., ZIEGLER, J. L., SIMON, R. & ANTHONY, P. P. (1975) A staging system for hepatocellular carcinoma—prognostic factors in Ugandan patients. *Cancer*, **35**, 1357.

PROVAN, J. L., STOKES, J. F. & EDWARDS, D. (1968) Hepatic artery infusion chemotherapy in hepatoma. *Br. Med. J.*, iii, 346.

PURVES, L. R., MACNAB, M. & BEERSOHN, I. (1968) Immunodiffusion and immunoassay results in cases of primary cancer. *S. Afr. Med. J.*, ii, 1138.

ROUSLATI, E., SEPPALA, M., VUOPIO, P., SAKSELA, E. & PELFOKALLIO, P. (1972) Radioimmunoassay of alpha-fetoprotein in primary and secondary cancer of the liver. *J. Natl Cancer Inst.*, **49**, 623.

VOGEL, C. I., PRIMACK, A., MCINTIRE, K. R., CARBONE, P. P. & ANTHONY, P. P. (1974) Serum alpha-fetoprotein in 184 Ugandan patients with hepatocellular carcinoma. *Cancer*, **33**, 959.